# The pulsing brain: part I

**DOI:** 10.1098/rsfs.2024.0045

**Published:** 2024-12-06

**Authors:** Andrea Lecchini-Visintini, Emma Chung, Samantha Holdsworth, Jatinder Minhas, Stephen Payne

**Affiliations:** ^1^ University of Southampton, Southampton, UK; ^2^ King’s College London, London, UK; ^3^ Matai Medical Research Institute & University of Auckland, New Zealand; ^4^ University of Leicester, Leicester, UK; ^5^ National Taiwan University, Taiwan

**Keywords:** brain tissue pulsation, mathematical modelling of biological tissues, mathematical modelling of biological fluids, MRI medical diagnostics, ultrasound medical diagnostics

This special issue contains invited contributions from the participants of a Royal Society Theo Murphy scientific meeting dedicated to ‘The Pulsing Brain’ held in June 2024 in Brighton, UK.

The pulsing brain is a complex dynamic phenomenon that necessitates interdisciplinary research yet presents significant opportunities to advance the understanding of disease progression and ultimately, brain health. The meeting gathered researchers from four key areas: magnetic resonance imaging (MRI) and ultrasound medical diagnostics, and mathematical modelling of biological tissues and fluids. The main objectives were to showcase existing data and models, highlight key research questions, explore potential pathways for clinical translation, foster new collaborations and ultimately to lay the foundation for a dedicated research community.

The invitation to contribute to this special issue was well received, resulting in a two-part issue, including a perspective paper, which will appear in Part II.

The contributions appearing in Part I are a balanced representation of all areas covered by the meeting, spanning MRI and ultrasound studies, modelling approaches and clinical applications.

The first two contributions of the issue provide novel insights obtained with MRI. van Hulst *et al*. [1] present a 7 T MRI study investigating local displacement of brain tissue during the cardiac cycle, revealing a heterogeneous volumetric strain map. Wright *et al*. [2] introduce a robust data-driven segmentation technique for cerebral vessels using pulsatile fMRI signals, facilitating the study of neurofluid dynamics.

The next two contributions illustrate the use of ultrasound technology in the context of brain pulsatility. Couvreur *et al*. [3] report on the link between elevated brain tissue pulsation and depression, illustrating how ultrasound-based measurements of tissue pulsatility can be used to form markers of brain health. Nicholls *et al.* [4] describe the development of transcranial tissue Doppler ultrasound, a novel modality for continuous monitoring of brain tissue pulsation.

The next contributions focus on the mathematical modelling of brain tissue and neurofluids. Greiner *et al.* [5] explore the complex mechanical behaviour of brain tissue specimens during loading and relaxation cycles using poro-viscoelastic models. Toscano *et al.* [6] use artificial intelligence velocimetry to quantify cerebrospinal fluid flow, incorporating uncertainty quantification to improve the accuracy of cerebrospinal fluid flow measurements.

The last two contributions of the issue illustrate the clinical context and novel applications. Iavarone *et al.* [7] review current neuromonitoring strategies for brain protection following cardiac arrest. Putnynaite *et al.* [8] report on the development of a novel non-invasive intracranial pressure monitoring device to improve neuromonitoring in traumatic brain injury and other neurological conditions.

Together, these contributions reflect the breadth of scope required to understand the pulsing brain and highlight the interdisciplinary approaches needed to gain new insights into brain health from this novel perspective.
